# Soshiho-Tang Aqueous Extract Exerts Antiobesity Effects in High Fat Diet-Fed Mice and Inhibits Adipogenesis in 3T3-L1 Adipocytes

**DOI:** 10.1155/2016/2628901

**Published:** 2016-09-29

**Authors:** Sae-Rom Yoo, Mee-young Lee, Byoung-Kab Kang, Hyeun-Kyoo Shin, Soo-Jin Jeong

**Affiliations:** ^1^K-Herb Research Center, Korea Institute of Oriental Medicine, Daejeon 34054, Republic of Korea; ^2^KM Fundamental Research Division, Korea Institute of Oriental Medicine, Daejeon 34054, Republic of Korea; ^3^KM Convergence Research Division, Korea Institute of Oriental Medicine, Daejeon 34054, Republic of Korea; ^4^Korean Medicine Life Science, University of Science & Technology, Daejeon 34113, Republic of Korea

## Abstract

Soshiho-tang (SST; sho-saiko-to in Japanese; xiaochaihu-tang in Chinese) has generally been used to improve liver fibrosis- and cirrhosis-related symptoms in traditional Korean medicine. Although many studies have investigated the pharmacological properties of SST, its antiobesity effect has not been elucidated. Thus, our present study was carried out to evaluate the antiobesity effect of SST using a high fat diet- (HFD) induced obese mouse model and 3T3-L1 adipose cells. C57BL/6J mice were randomly divided into four groups (*n* = 6/group), normal diet (ND), HFD-fed group, and HFD- and SST-fed groups (S200: 200 mg/kg of SST; S600: 600 mg/kg of SST) and given HFD with or without SST extract for 8 weeks. 3T3-L1 preadipocytes were differentiated into adipocytes for 8 days with or without SST. In the HFD-fed obese mice, body weight and fat accumulation in adipose tissue were significantly reduced by SST administration. Compared with control-differentiated adipocytes, SST significantly inhibited lipid accumulation by decreasing the triglyceride (TG) content and leptin concentration in 3T3-L1 adipocytes. SST also decreased the expression of adipogenesis-related genes including lipoprotein lipase (LPL), fatty acid binding protein 4 (FABP4), CCAAT/enhancer-binding protein-alpha (C/EBP-*α*), and peroxisome proliferator-activated receptor-gamma (PPAR-*γ*). Our findings suggest that SST has potential as a nontoxic antiobesity medication.

## 1. Introduction

Obesity or overweight is abnormal or excessive fat accumulation resulting from an energy imbalance. Obesity is a key risk factor for various diseases, such as type 2 diabetes, cardiovascular disease, nonalcoholic fatty liver disease, and cancer [1]. According to Ogden's report, more than one-third (34.9%) of adults in the United States are obese and the proportion is continuously increasing [2]. The number of obese people in South Korea has also been rising since 2001, reaching approximately 32% in 2012 [3]. To date, several medications for obesity treatment have been approved by the Food and Drug Administration (FDA), such as orlistat (Xenical), lorcaserin, and the phentermine/topiramate (Qsymia) drug combination [4]. Although they are effective in reducing body weight or intestinal fat absorption, severe side effects remain problematic. Thus, the development of antiobesity drugs with better efficacies and fewer side effects is necessary.

Obesity is associated with adipogenesis, the cell differentiation process from preadipocytes into adipocytes [5]. The molecular pathway of adipogenesis is regulated by various transcription factors, such as peroxisome proliferator-activated receptor-gamma (PPAR-*γ*) and CCAAT/enhancer-binding protein-alpha (C/EBP-*α*) [6], and adipokines, the adipocyte-secreted cytokines. Targeting adipogenesis is considered to be one of the more favored approaches to controlling obesity. Indeed, several groups have reported the antiobesity potentials of herbal medicines that prevent adipogenesis [7–9].

In the traditional Korean medicine (TKM), obesity is mainly caused by phlegm, dampness, and stagnated blood and its treatment is conducted by promoting blood flow to remove blood stasis and resolving phlegm or dampness [10]. Soshiho-tang (SST; xiaochaihu-tang in Chinese; sho-saiko-to in Japanese) is a traditional herbal formula comprising seven different medicinal herbs and has been used to treat chronic liver disease. Pharmacological properties of SST in the TKM are to clear up heat and resolve dampness in liver, spleen, and stomach according to the Sanghan-ron (150–219 AD in the Chinese Eastern Han Dynasty). SST has generally been used to improve liver fibrosis- and cirrhosis-related symptoms such as fatigue, nausea, and inappetence in traditional Korean medicine. Recent pharmacological and clinical studies demonstrated that SST has the efficacies of immunomodulation [11–13] and hepatoprotection [14–16]. Importantly, our group reported on the safety of SST by subacute and subchronic toxicity tests using Sprague Dawley rats [17, 18]. The SST was administered once daily to both sexes of SD rats at dose of 2000 mg/kg/day for 4 or 13 weeks. The SST treatment did not result in any toxicological changes in clinical signs including mortality, body weight, food consumption, gross findings, hematological and biochemical parameters, ophthalmoscopy, and urinalysis. When rats were orally treated with SST administered for 13 weeks, there were no histopathological findings in the kidney and liver [17].

However, there is no scientific evidence that SST has an antiobesity effect. In the present study, we investigated the antiobesity effect of SST using* in vitro* and* in vivo* models.

## 2. Materials and Methods

### 2.1. Plant Materials and Preparation of SST

The seven herbal components of SST were purchased from HMAX (Jecheon, Korea) and Omniherb (Yeongcheon, Korea) (Table 1). A ground herbal medicines mixture of 10.0 kg was extracted in a 10-fold volume of water at 100°C for 2 h under pressure (1 kgf/cm^2^) using an electric extractor (COSMOS-660; Kyungseo Machine Co., Incheon, Korea). The water extract was then filtered through a standard sieve (number 270, 53 *μ*m; Chung Gye Sang Gong Sa, Seoul, Korea) and the solution was freeze-dried to a powder (Innova® U725 Upright Freezer, Eppendorf, Hamburg, Germany). The yield of SST extract was 16.37%, and it was stored below 4°C. We dissolved the power in DPBS, and then the stock solution was filtered through a 0.2 *μ*m pore membrane filter. Voucher specimens (2008-KE26-1-12) have been deposited at the Herbal Medicine Formulation Research Group, Korea Institute of Oriental Medicine. High-performance liquid chromatography (HPLC) analysis data for the quality control of SST have been provided in our previous reports [17, 18].

### 2.2. Animals, Diet, and Experimental Design

Six-week-old C57BL/6J mice were purchased from Central Lab. Animal Inc. (Seoul, Korea). After allowing one week for adaptation, the mice were randomly assigned into 4 groups (*n* = 6/group): normal diet (ND; 10% fat, Research Diets, Inc., New Brunswick, NJ), high fat diet-fed group (HFD; 60% fat, Research Diets, Inc.), HFD with 200 mg/kg of SST (S200), and HFD with 600 mg/kg of SST (S600). Starting from 2 weeks after HFD feeding, SST extract was administered for 8 weeks. Food intake and body weight were monitored twice a week. The compositions of ND and HFD are shown in Table 2. After 8 weeks of SST treatment, the mice were fasted for 12 h and then sacrificed under anesthesia. Blood was taken from the right ventricle and collected in an EDTA-coated tube. Liver and adipose tissue were dissected and weighed and stored at −80°C. All experimental procedures involving animals were conducted in accordance with the NIH Guidelines for the Care and Use of Laboratory Animals and approved by the Korea Institute of Oriental Medicine Institutional Animal Care and Use Committee (Approval number 13-090).

### 2.3. Measurement of Serum Triglyceride

Serum triglyceride (TG) was enzymatically determined using a commercial kit (Asan Co., Seoul, Korea). Briefly, serum was mixed with lipase and a TG reaction mixture at 37°C for 5 min. The absorbance was then measured at 550 nm using a microplate reader (Benchmark Plus Microplate spectrophotometer, Bio-Rad Laboratories, Hercules, CA).

### 2.4. Measurement of Serum Total Cholesterol

Serum total cholesterol was measured using a total cholesterol quantitation kit (Asan Co., Seoul, Korea). Cholesterol esterase was added to each serum to hydrolyze and free cholesterol and fatty acids, which were then oxidized by cholesterol oxidase to yield H_2_O_2_ and *δ*4-cholestenone. Adding a reaction mixture generated quinoneimine dye, which was determined using a microplate reader (as above) at 500 nm.

### 2.5. 3T3-L1 Cell Differentiation and SST Treatment

3T3-L1 (ATCC CL-173) was purchased from the American Type Culture Collection (ATCC, Rockville, MD) and maintained in Dulbecco's modified Eagle's medium (DMEM) supplemented with 10% newborn calf serum (Gibco, Carlsbad, CA).

The cells were cultured in DMEM supplemented with 10% newborn calf serum (Gibco) at 37°C. For adipocyte differentiation, the cells were stimulated with 3T3-L1 differentiation medium containing isobutylmethylxanthine, dexamethasone, and insulin (MDI) (Zen-Bio Inc., Research Triangle Park, NC) for 48 h after reaching a confluent state. The medium was switched to DMEM containing 10% FBS and 1 *μ*g/mL insulin after 2 days and then changed to DMEM containing 10% FBS for an additional 4 days. SST extract was added to the cell culture during the 8 days of differentiation. GW9662 (Sigma-Aldrich, St. Louis, MO), a PPAR-*γ* antagonist, was used as a positive control.

### 2.6. Cytotoxicity Assay

Preadipocytes and adipocytes were exposed to various concentrations of SST for 24 h or 8 days (differentiation period), respectively. CCK-8 solution (Dojindo Lab, Tokyo, Japan) was added to the cells, which were then incubated for 1 h. Cell viability was calculated as the percentage of viable cells in the SST-treated cells versus untreated cells.

### 2.7. Oil-Red O (ORO) Staining

ORO staining was assayed according to the modified previous method [19, 20]. The differentiated adipocytes were washed twice with PBS and fixed with 10% formaldehyde for 1 h at room temperature. After fixation, the cells were washed with 70% ethanol and PBS and stained with ORO (Sigma-Aldrich) for 30 min. Photographs of stained cells were taken with an Olympus CKX41 inverted microscope (Olympus, Tokyo, Japan). To quantify fat accumulation, the cells were dissolved in isopropyl alcohol and measured by reading the absorbance at 530 nm (Benchmark Plus Microplate Spectrophotometer, Bio-Rad Laboratories).

### 2.8. TG Quantification Assay

The TG concentration was measured enzymatically using a commercial kit (BioVision Inc., Milpitas, CA). Briefly, the cells were homogenized in 5% NP-40 assay buffer, and the homogenized cells were heated slowly to solubilize all TGs. And then it was mixed with lipase and TG reaction mixture. After 1 h incubation, the sample absorbance was measured at 570 nm using a microplate reader (Benchmark Plus, Bio-Rad Laboratories).

### 2.9. *In Vitro* and* In Vivo* Leptin Immunoassay

Leptin concentration was measured using a mouse leptin immunoassay kit (R&D Systems, Minneapolis, MN) in accordance with the manufacturer's instructions. After loading cell culture supernatant or mouse plasma samples, equal amounts of the supernatants (50 *μ*L) and Assay Diluent RD1W (50 *μ*L) were added to 96-well plates, which were incubated for 2 h at room temperature. The plates were washed 5 times with 400 *μ*L of wash buffer, 100 *μ*L of mouse leptin conjugate was added to each well, and they were incubated for 2 h at room temperature. The plates were washed 5 times, 100 *μ*L of substrate solution was added to each well, and they were incubated for 30 min at room temperature in the dark. Finally, 100 *μ*L of stop solution was added to each well, and the absorbance was measured at 450 nm using a microplate reader (Benchmark Plus, Bio-Rad Laboratories).

### 2.10. RNA Isolation and RT-PCR

Total RNA was isolated using TRIZOL reagent (Invitrogen, Carlsbad, CA) in accordance with the manufacturer's instructions. The total RNA was synthesized to cDNA using an iScript cDNA synthesis kit (Bio-Rad Laboratories) and subjected to PCR reaction with rTaq DNA polymerase (ELPIS Biotech Inc., Daejeon, South Korea). The conventional PCR conditions were 22–28 cycles at 94°C for 30 sec, 50–60°C for 1 min, and 72°C for 1.5 min. The amplification products were separated by electrophoresis on 1% agarose gels and detected with a Molecular Imager® Gel Doc*™* XR System (Bio-Rad Laboratories).

### 2.11. Protein Extraction and Western Blotting

After differentiation, 3T3-L1 cells were washed twice with ice-cold PBS. The cells were then lysed in lysis buffer containing protease inhibitor (Roche Applied Science, Indianapolis, IN). The lysates were centrifuged at 14,000 ×g for 15 min at 4°C, and the protein concentrations in the supernatants were determined using Bradford Reagent (Bio-Rad Laboratories). Western blotting was performed with precast gels (Bio-Rad Laboratories) and all separated proteins were transferred to polyvinylidene difluoride membranes (Amersham Biosciences, Piscataway, NJ). The membranes were blocked with 5% (w/v) nonfat dry milk for 1 h and then each membrane was incubated with primary antibodies for 24 h at 4°C. After the removal of the primary antibody, the membranes were washed three times with Tris-buffered saline-Tween (TBST) buffer and incubated with horseradish peroxidase-conjugated secondary antibody (Jackson Immunoresearch, West Grove, PA) for 1 h at room temperature. The immunoreactive bands were visualized with ECL reagent (Thermo Scientific, Rockford, IL).

### 2.12. Statistical Analysis

All data are presented as mean ± the standard error of the mean (SEM). In* in vitro* data, group differences were assessed by one-way ANOVA and* post hoc* Tukey's multiple comparison test using GraphPad InStat ver. 3.10 (GraphPad Software, Inc., San Diego, CA). In* in vivo* data, significant differences between all groups were assessed by one-way ANOVA and Duncan's multiple range test using SPSS ver. 17 (SPSS Inc., Chicago, IL).

## 3. Results

### 3.1. Inhibitory Effect of SST on Lipid Accumulation and Leptin Production in 3T3-L1 Adipocytes

Cytotoxicity of SST water extract was evaluated in the undifferentiated and differentiated 3T3-L1 cells. SST has no toxicity against preadipocytes up to 500 *μ*g/mL treatment (Figure 3(a)). In the adipocytes, SST treatment had no cytotoxic effect up to 250 *μ*g/mL but reduced the cell viability by 65.07% at 500 *μ*g/mL treatment (Figure 3(b)). Thus, nontoxic concentrations of SST were used for all subsequent experiments using 3T3-L1 adipocytes.

To test the antiadipogenic effect of SST, 3T3-L1 adipocytes were treated with SST extract during 8 days of the differentiation period. As shown in Figure 4(a), the number of lipid droplets was markedly detectable in the adipocytes compared with the preadipocytes. In contrast, SST treatment reduced fat accumulation of the adipocytes compared with untreated cells (Figure 4(a)). Adipose tissue is the major storage site for lipids in the form of TG. For this reason, the morphological observations shown in Figure 4(a) were confirmed by assessing the TG contents in adipocytes. Consistent with the results of ORO staining, the TG content was significantly decreased by SST treatment in a dose-dependent manner compared with untreated adipocytes (Figure 4(b)). Additionally, SST administration significantly decreased the amount of leptin, one of major adipogenesis markers, at 100 or 200 *μ*g/mL, but not at 50 *μ*g/mL (Figure 4(b)). GW9662 was used as a positive control.

### 3.2. Suppressive Effects of SST on Expression of Adipogenesis-Related Biomarkers in 3T3-L1 Adipocytes

To understand the molecular mechanisms responsible for the antiadipogenic activity of SST, expression of the biomarkers involved in the adipogenesis pathway was analyzed in SST-treated 3T3-L1 adipocytes at the protein and mRNA levels. As shown in Figure 5(a), protein expressions of PPAR-*γ* and C/EBP-*α*, major transcriptional factors in adipogenesis, were markedly enhanced in adipocytes compared with preadipocytes. In contrast, SST treatment reversed the adipogenesis-mediated increase of PPAR-*γ* and C/EBP-*α* expression compared with untreated adipocytes. SST treatment also downregulated protein levels of fatty acid synthase (FAS), perilipin, and fatty acid binding protein 4 (FABP4) corresponding with PPAR-*γ* expression (Figure 5(b)).

Consistent with the results of immunoblotting, SST treatment suppressed mRNA levels of PPAR-*γ* and C/EBP-*α*, FAS, FABP4, and lipoprotein lipase (LPL) in 3T3-L1 adipocytes (Figure 6).

### 3.3. Effect of SST on Fat Accumulation in Adipose Tissue of HFD-Fed Obese Mouse Model

**Figure 1:**
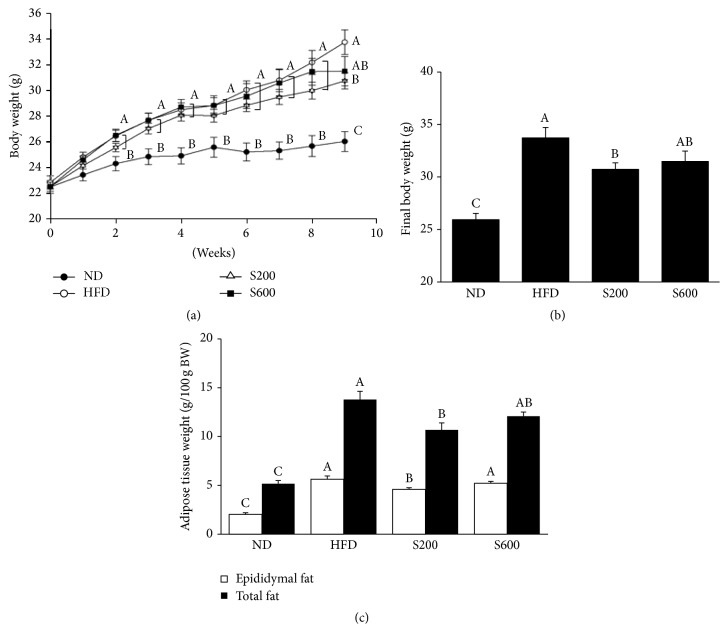
Effects of SST extract on (a) body weight change, (b) final body weight, and (c) adipose tissue weight. Values with the different superscript letters indicate statistical significance (*p* < 0.05) between groups by Duncan's multiple range test. ND: normal diet-fed group, HFD: high fat diet-fed group, S200: HFD-fed group with 200 mg/kg of SST, and S600: HFD-fed group with 600 mg/kg of SST.

C57BL/6J mice were fed with HFD with or without oral administration of SST for 8 weeks at 200 or 600 mg/kg/day. Body weight was significantly increased in the HFD-fed group compared with the ND-fed group. SST administration showed significantly reduced body weight at 200 mg/kg, but not 600 mg/kg, compared with the HFD-fed group (Figure 1(a)). Consistently, HFD feeding significantly increased total fat from 5.1683 to 13.7914 g/100 g body weight and epididymal fat pad weight from 2.0233 to 5.6312 g/100 g body weight. In contrast, SST administration at 200 mg/kg significantly decreased the total and epididymal fat weight from 13.7914 to 10.6838 g/100 g body weight and from 5.6312 to 4.5898 g/100 g body weight, respectively. However, SST at 600 mg/kg had no significant effect on the HFD group (Figure 1(b)).

### 3.4. Effect of SST on Plasma Lipid Levels in HFD-Fed Obese Mouse Model

**Figure 2:**
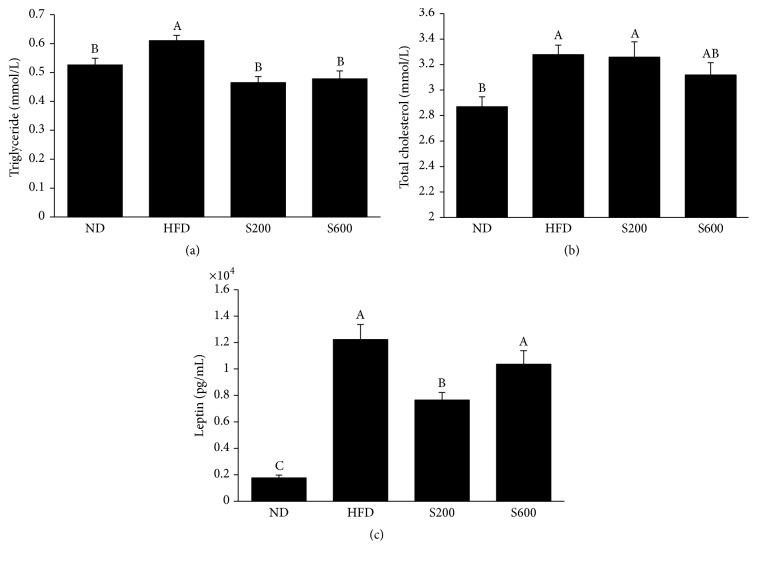
Effects of SST on plasma levels of (a) TG, (b) total cholesterol, and (c) leptin production in HFD-fed mice. Values with the different superscript letters indicate statistical significance (*p* < 0.05) between groups by Duncan's multiple range test. ND: normal diet-fed group, HFD: high fat diet-fed group, S200: HFD-fed group with 200 mg/kg of SST, and S600: HFD-fed group with 600 mg/kg of SST.

**Figure 3:**
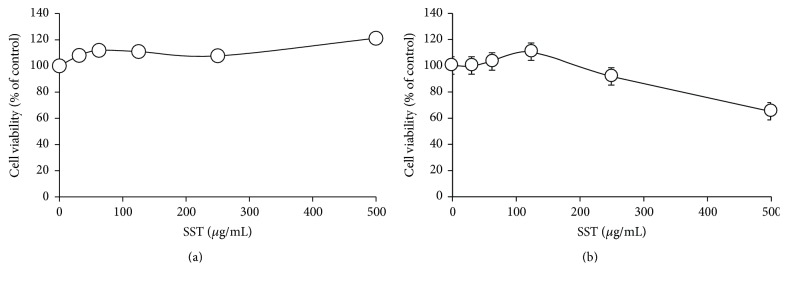
Cytotoxic effects of SST in (a) preadipocytes and (b) adipocytes. (a) 3T3-L1 preadipocytes were treated with various concentrations of SST for 24 h. (b) 3T3-L1 preadipocytes were differentiated into adipocytes by incubation with isobutylmethylxanthine, dexamethasone, and insulin (MDI) for 8 days. The cells were exposed to various concentrations of SST during the differentiation period. Data are presented as mean ± SEM.

**Figure 4:**
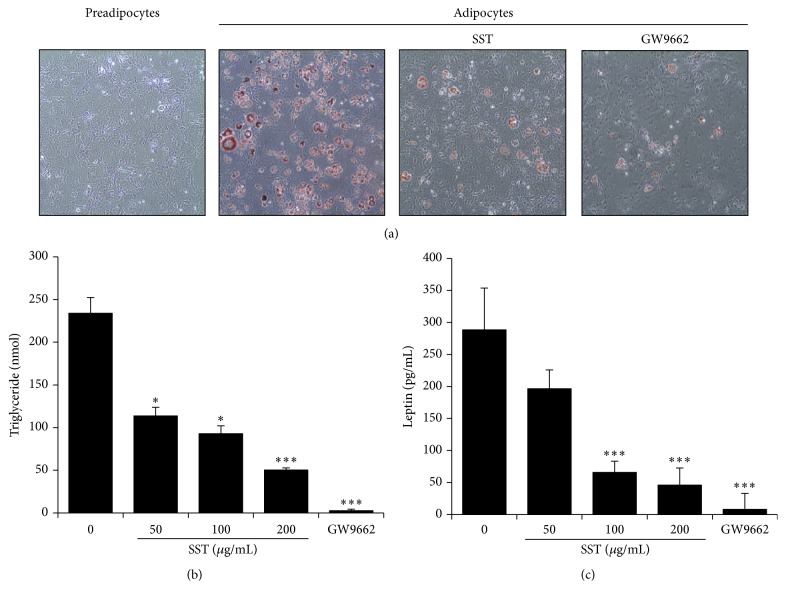
Inhibitory effect of SST extract on TG production in 3T3-L1 adipocytes. 3T3-L1 preadipocytes were differentiated into adipocytes by incubation with isobutylmethylxanthine, dexamethasone, and insulin (MDI) for 8 days. The cells were treated with or without SST or GW9662 (20 *μ*M) during the differentiation period. (a) Lipid accumulation in the cells was analyzed by Oil-Red O staining. (b) The TG content was measured enzymatically using a commercial kit. (c) Culture supernatant was collected from the SST-treated cells. Leptin production was determined by ELISA by using a mouse leptin immunoassay kit. Data are presented as mean ± SEM. ^*∗*^ *p* < 0.05 and ^*∗∗∗*^ *p* < 0.001* versus* differentiated cells. GW9662 (20 *μ*M) was used as a positive control.

**Figure 5:**
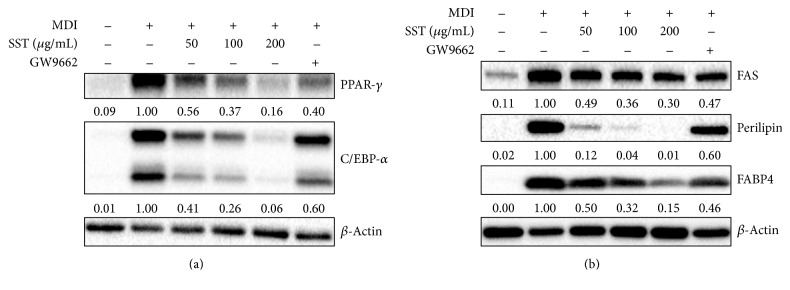
Effects of SST on protein expression of lipid metabolism-related genes in 3T3-L1 adipocytes. 3T3-L1 adipocytes were exposed to various concentrations of SST or GW9662 (20 *μ*M) during the differentiation period. Cell lysates were prepared and subjected to Western blotting for PPAR-*γ* and C/EBP-*α* (a) and FAS, perilipin, and FABP4 (b). *β*-Actin was used as a loading control.

**Figure 6:**
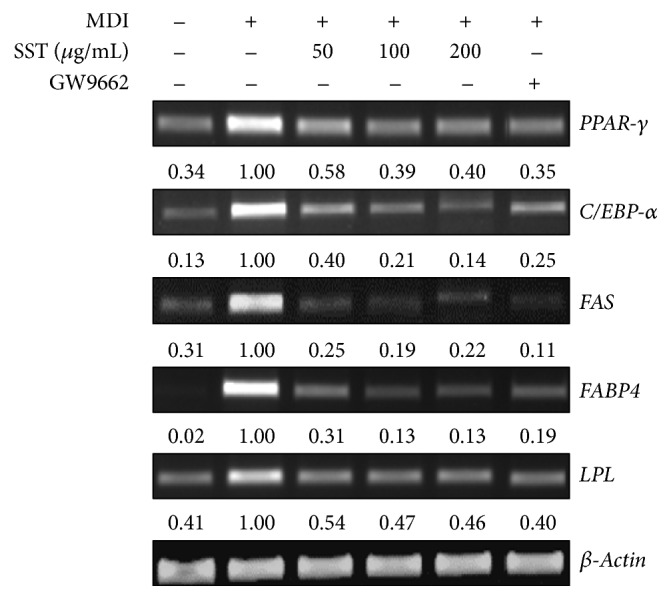
Effects of SST on mRNA expression of lipid metabolism-related genes in 3T3-L1 adipocytes. 3T3-L1 adipocytes were exposed to various concentrations of SST or GW9662 (20 *μ*M) during the differentiation period. Total RNA was isolated and subjected to RT-PCR for* FASN*,* FABP4*,* LPL*,* C/EBP-α*,* and PPAR-γ*. *β*-Actin was used as a housekeeping gene.

Levels of TG and total cholesterol were assessed in plasma samples from the four different groups. As shown in Figures 2(a) and 2(b), plasma TG and total cholesterol levels were significantly increased in HFD-fed mice. In contrast, SST treatment at 200 or 600 mg/kg led to a marked reduction in plasma TG levels (Figure 2(a)) but had no effect on plasma total cholesterol level (Figure 2(b)). In addition, leptin production in plasma was measured by ELISA. Consistent with the results of adipose tissue weight, HFD significantly increased the level of leptin compared with the normal diet, whereas SST administration at 200 mg/kg reduced HFD-induced leptin production compared with the HFD control group (Figure 2(c)).

## 4. Discussion

Herbal medicines have increased in popularity in recent decades as an alternative way of reducing the adverse effects of Western medicines. Herbal formula, a mixture of several herbs, has been used for centuries to treat a range of diseases in Asian countries. From a practical perspective, the practitioner combines herbs based on the patient's diagnosis and then prepares a decoction. Recently, many researchers have investigated the effectiveness and safety of herbal formulas to provide scientific evidence for them.

Several groups have reported that traditional herbal formulas have potent effects as antiobesity agents. Hwangryunhaedok-tang (Orengedokuto) inhibited differentiation and lipid accumulation in 3T3-L1 adipocytes [21, 22] and lowered body weight and waist circumstance in abdominally obese patients [9]. Bangpungtongseong-san (Bofu-tsusho-san) [23] and Dohaekseunggi-tang [24] attenuated metabolic disorders, including hypertension and visceral obesity.

In the present study, we demonstrated that water extract of SST has antiobesity activity using* in vitro* and* in vivo* models. We previously reported HPLC analysis of SST. To calculate the contents of the main components in SST, we applied it to the simultaneous analysis of three compounds: liquiritin, baicalin, and glycyrrhizin. Among them, the baicalin showed a maximum peak height. The contents of three components did not show significant differences during storage periods [17, 18]. In another report, Yang et al. reported that the efficient HPLC coupled with diode array detection and electrospray ionization tandem mass spectrometry (HPLC-DAD-ESI-MS) was applied for simultaneous analysis of the six compounds: homogentisic acid, baicalin, glycyrrhizin, saikosaponin A, 6-gingerol, and ginsenoside Rg3 [25]. Although the baicalin showed a maximum peak height for those compounds, the other showed a small peaks. In contrast, we found two peaks except the baicalin in HPLC chromatogram of SST. Under optimized chromatographic conditions, the separation time was different.

SST consists of seven medicinal herbs: Bupleuri Radix, Scutellariae Radix, Ginseng Radix, Pinelliae Tuber, Zingiberis Rhizoma Crudus, Zizyphi Fructus, and Glycyrrhizae Radix et Rhizoma. Among these components, baicalin [26, 27] and baicalein [28] from Scutellariae Radix and ginsenoside Rg1 from Ginseng Radix Alba [29] are well known for antiadipogenic effects. Although a previous study indicated that SST suppresses epididymal fat weight and altered serum lipid profile in high fat-fed rats [30], the regulatory mechanisms responsible for the antiobesity effect of SST have not been elucidated. In HFD-fed mice, oral gavage of SST extract (200 mg/kg) reduced final body weight and adipose tissue weights in a diet-induced obese mouse model. The main role that adipose tissue plays is in whole-body energy homeostasis and lipid storage as triglycerides, which can rapidly respond to energy imbalance [31]. SST administration significantly decreased the levels of hyperlipidemic parameters such as TG and total cholesterol, as well as the production of leptin, an adipokine secreted during adipocyte differentiation [32], in the serum of HFD-fed mice, which is consistent with the results of Haeng et al. [30].

When energy intake is higher than energy expenditure, it leads to increased adipocyte hyperplasia and hypertrophy through adipogenesis, thereby accelerating the obese state. Thus, we investigated the antiobesity effects of SST using 3T3-L1 adipocytes, commonly used as an* in vitro* model system to study the molecular mechanisms of adipogenesis [33]. SST extract reduced the formation of lipid droplets in mature adipocytes compared with the differentiated group. During the late stage of differentiation, TGs accumulate in adipocytes through fatty acid synthesis in the cytoplasm [34]. For this reason, we assessed whether SST can alter the TG content in adipocytes. SST treatment resulted in reduction of the intracellular TG content [35]. In addition, secreted amounts of leptin appeared to be reduced by SST treatment, consistent with the results of the* in vivo* experiment.

Adipocyte differentiation is regulated by a cascade of multiple transcriptional factors [36]. PPAR-*γ* and C/EBP-*α*, master adipogenic transcription factors, are involved in the terminal stage of the adipogenesis process and control the expression of target genes leading to adipocyte development [37, 38]. In the current study, treatment with SST extract markedly decreased expression of PPAR-*γ* and C/EBP-*α* at the mRNA and protein levels in 3T3-L1 adipocytes. Furthermore, SST reduced the adipogenesis-mediated increase in the protein and mRNA levels of the PPAR-*γ* target molecules.

Our data demonstrate that SST extract reduced body weight and adipose tissue weight in HFD-fed mice. SST also inhibited adipogenesis in 3T3-L1 adipocytes by decreasing TG accumulation and cascade of multiple transcriptional factors including PPAR-*γ* and C/EBP-*α* at the mRNA and protein levels. These findings suggest that SST can be considered a potential drug candidate for the treatment of obesity.

## Figures and Tables

**Table 1:** Composition of SST.

Latin name	Amount (g)	Ratio	Supplier	Source
Bupleuri Radix	11.25	6	HMAX	China
Pinelliae Tuber	7.5	4	HMAX	Jeongseon, Korea
Scutellariae Radix	3.75	2	HMAX	China
Ginseng Radix Alba	3.75	2	Omniherb	Geumsan, Korea
Zizyphi Fructus	3.75	2	Omniherb	Yeongcheon, Korea
Zingiberis Rhizoma Crudus	3.75	2	Omniherb	Yeongcheon, Korea
Glycyrrhizae Radix	1.875	1	HMAX	China

Total	35.625			

**Table 2:** Composition of experimental diet.

	ND		HFD	
	gm%	kcal%	gm%	kcal%
Protein	19.2	26	24	20
Carbohydrate	67.3	70.0	26	20
Fat	4.3	10.0	35	60
Total		100.0		100
kcal/gm	3.85		5.24	

Ingredient	gm	kcal	gm	kcal

Casein, 80 mesh	200	800	200	800
L-Cystine	3	12	3	12

Corn starch	315	1260	0	0
Maltodextrin 10	35	140	125	500
Sucrose	350	1400	68.8	275

Cellulose	50	0	50	0

Soybean oil	25	225	25	225
Lard	20	180	245	2205

Mineral mix S10026	10	0	10	0
Dicalcium phosphate	13	0	13	0
Calcium carbonate	5.5	0	5.5	0
Potassium citrate	16.5	0	16.5	0

Vitamin mix, V1001	10	40	10	40
Choline bitartrate	2	0	2	0

Total	1055.05	4057	773.85	4057

ND: normal diet group; HFD: high fat diet group.
